# Intrinsic circadian organization of the spinal cord and dorsal root ganglia

**DOI:** 10.1097/PR9.0000000000001505

**Published:** 2026-10-08

**Authors:** Shintaro Yamazaki, Lee Wulund, Utham K. Valekunja, Akhilesh B. Reddy

**Affiliations:** a Department of Systems Pharmacology & Translational Therapeutics, Perelman School of Medicine, University of Pennsylvania, Philadelphia, PA, USA. Dr. Valekunja is now with the National Institute of Animal Biotechnology (NIAB), Hyderabad, Telangana, India; b Institute for Translational Medicine and Therapeutics, Perelman School of Medicine, University of Pennsylvania, Philadelphia, PA, USA; c Chronobiology and Sleep Institute (CSI), Perelman School of Medicine, University of Pennsylvania, Philadelphia, PA, USA; d University of Cambridge Metabolic Research Laboratories, Wellcome Trust-MRC Institute of Metabolic Science, Addenbrooke's Hospital, Cambridge, United Kingdom

**Keywords:** Circadian rhythms, Spinal cord, Dorsal root ganglia, Somatosensory system, Primary sensory neurons, Circadian clock, Temporal gene regulation, Sensory signaling, Transcriptomics, Chronotherapy, Pain modulation, RNA sequencing

## Abstract

**Introduction:**

Circadian rhythms coordinate daily fluctuations in physiology and behavior, yet their organization within primary sensory pathways remains poorly defined. Although somatosensory responsiveness varies across the day–night cycle, it is unclear whether peripheral sensory circuits possess molecular mechanisms for temporal regulation.

**Objectives:**

To determine whether the spinal–peripheral sensory axis harbors intrinsic circadian clocks and to define the molecular scope and functional organization of circadian regulation in dorsal root ganglia (DRGs).

**Methods:**

We used real-time bioluminescence imaging to assess circadian oscillations of the core clock protein PER2 in the spinal dorsal horn and DRGs. To characterize molecular regulation, we performed RNA sequencing across a 52-hour circadian time course in DRGs.

**Results:**

We demonstrate that the spinal dorsal horn and DRGs harbor robust, tissue-autonomous circadian clocks, with sustained PER2 oscillations. Circadian analysis identified 626 rhythmic genes (3.6% of expressed genes) in DRGs, with nonuniform phase distributions forming discrete temporal clusters. Functional annotation revealed phase-specific enrichment of processes including transport, neuronal structure, and proteostasis, suggesting coordinated temporal deployment of distinct molecular programs rather than uniform oscillations. Cross-referencing with neuropathic pain-associated genes revealed limited overlap; however, overlapping genes aligned to specific baseline phase windows enriched for regenerative annotations. Potassium channel-related signaling components implicated in neuropathic pain also showed baseline circadian modulation.

**Conclusion:**

These findings establish the spinal dorsal horn and DRGs as intrinsically circadian tissues and reveal a temporally structured molecular landscape in DRG, providing a framework for understanding how peripheral sensory processing, plasticity, and homeostatic regulation are coordinated across the day–night cycle.

## Abstract 2

Supplemental Digital Content is Available in the Text.

The spinal dorsal horn and dorsal root ganglia harbor robust circadian clocks, and the DRG organizes daily gene programs linked to sensory signaling, plasticity, and cellular maintenance.

## 1. Introduction

Circadian rhythms are near-24-hour oscillations coordinating molecular, cellular, and physiological processes. In mammals, these rhythms arise from cell-autonomous transcription–translation feedback loops involving core clock genes, while systemic synchronization is coordinated by the suprachiasmatic nucleus (SCN).^27,63^ Although circadian regulation has been described in many peripheral tissues and brain regions, its organization within the primary sensory axis remains incompletely defined.^63^

The primary somatosensory system lies at the interface between the external environment and the central nervous system. Peripheral sensory information is conveyed by primary afferents whose cell bodies reside in the dorsal root ganglia (DRGs), with axons projecting to peripheral targets and centrally to the spinal dorsal horn.^34^ Dorsal root ganglia neurons comprise molecularly distinct subtypes specialized for mechanosensation, thermosensation, proprioception, and nociception.^2,19,43,50^

Temporal variation in somatosensory responsiveness has long been recognized. Human studies report diurnal fluctuations in pain sensitivity and analgesic efficacy,^6,8,25,36,61,76^ and rodent studies show circadian modulation of thermal and mechanical sensitivity, including in neuropathic and chemotherapy-induced pain models.^12,15,37,38,40,41,53^ Rhythmic variation persists after nerve injury,^80^ suggesting that time-of-day effects are not explained solely by behavioral state or environmental cues. At the molecular level, pain-relevant pathways also show daily variation, including rhythmic *Tac1* expression in spinal dorsal horn and diurnal changes in the α2δ-1 calcium channel subunit and gabapentin binding in DRGs after partial sciatic nerve ligation.^41^

Despite this evidence, it remains unclear whether temporal variation in sensory processing reflects intrinsic circadian organization of the primary sensory pathways. In particular, autonomous circadian clocks within spinal cord and DRGs have not been systematically characterized. Here, we investigated circadian clock function in mouse spinal dorsal horn and DRGs using bioluminescence imaging and transcriptomic profiling. Using *mPer2*^*luc*^ reporter mice, we assessed intrinsic circadian oscillations and defined the molecular landscape of circadian regulation in adult male DRGs by RNA-seq across a circadian time course.^83^ Together, these approaches show that the spinal–peripheral sensory axis harbors autonomous circadian clocks and a phase-structured DRG transcriptome.

## 2. Methods

### 2.1. Animals

All animal procedures were conducted in accordance with the United Kingdom Animals (Scientific Procedures) Act 1986, and approved by the United Kingdom Home Office and the local ethical review panel at the University of Cambridge. Adult and postnatal female and male C57Bl/6J and *mPer2*^*Luc*83^ reporter mice were used in this study. Unless otherwise stated, mice were maintained under controlled conditions with ad libitum access to food and water on a 12 hours:12 hours light–dark (LD) cycle. Ambient temperature was maintained at 22 ± 2°C.

### 2.2. Organotypic tissue culture and bioluminescence recordings

Organotypic tissue cultures were prepared from adult and postnatal (P7-P10) *mPer2*^*Luc*^ reporter mice. Animals were culled by cervical dislocation and tissues rapidly dissected into cold Gey's Balanced Salt Solution (Sigma-Aldrich, St Louis, MO) supplemented with 5.0 g/L glucose, 100 nM MK-801, 50 μM DL-AP5 (Tocris Bioscience, Bristol, United Kingdom), and 3 mM MgCl2 (Sigma-Aldrich). Spinal cords were isolated and sectioned transversely (300 µm) using a McIlwain tissue chopper (Mickle Laboratory Engineering, Guildford, United Kingdom). Suprachiasmatic nucleus slices were prepared coronally (200 µm) using a vibrating microtome (7000smz-2, Campden Instruments, Loughborough, United Kingdom). For rostrocaudal comparisons, spinal cords were bisected along the midline and cultured intact. Adult mouse DRGs were dissected using fine forceps with proximal and distal axons severed and cultured intact. For serum shock experiments, cervical DRG explants were exposed to 50% horse serum for 2 hours, and phase shifts were calculated from the first post-treatment PER2LUC peak.

Tissues were placed on culture membranes (Merck Millipore, Watford, United Kingdom) and maintained in sealed culture dishes containing modified air medium composed of DMEM (Sigma-Aldrich) supplemented with 30 mM glucose, 20 mM HEPES, 0.035% NaHCO_3_, 25% (v/v) heat-inactivated horse serum (Thermo Fisher Scientific, Waltham, MA), 1× GlutaMAX, 1× B-27 supplement, 1 mM luciferin (Biosynth, Staad, Switzerland), and 1× MycoZap Plus-PR (Lonza, Basel, Switzerland).

Bioluminescence recordings were performed using a custom imaging system (Cairn Research Ltd, Faversham, United Kingdom) equipped with an Andor iKon-M 934 CCD camera (Andor Technology, Belfast, United Kingdom) housed within a Galaxy 170R incubator (Eppendorf, Hamburg, Germany). Temperature control and image acquisition were managed with MetaMorph software (Molecular Devices, Sunnyvale, CA). Images were acquired with 25-minute exposures at 30-minute intervals. Image stacks were compiled in ImageJ (NIH, Bethesda, MD), and bioluminescence signals were quantified from manually defined regions of interest. Integrated density values were extracted and analyzed using the CellulaRhythm R script.^29^

High-magnification (10×) bioluminescence imaging of spinal cord sections was performed using a Hamamatsu ImageEM 1K camera (Hamamatsu Photonics, Hamamatsu, Japan) mounted on an Eclipse Ti-E microscope (Nikon, Tokyo, Japan). Culture membranes were transferred to 35-mm glass-bottom dishes and maintained in a temperature-controlled incubation chamber (37°C; Okolab, Pozzuoli, Italy). Images were acquired every 30 minutes using NIS Elements AR software (version 4; Nikon), exported to ImageJ, and analyzed as described above.

### 2.3. Circadian entrainment and RNA isolation

Circadian entrainment was performed in ventilated housing cabinets (Tecniplast, United Kingdom) with lighting controlled by ClockLab software (Actimetrics, Wilmette, IL). Adult male C57Bl/6J mice were entrained to LD cycle for 2 weeks, released into constant darkness (DD) for 2 days, and culled by cervical dislocation on the third day in DD at 4-hour intervals from circadian time 0 (CT0; n = 3) to CT52. CT0 was defined as the projected lights-on time from the preceding LD cycle.

Cervical DRGs were rapidly dissected, homogenized in TRI Reagent (Zymo Research), snap-frozen, and stored at −80°C. Total RNA was extracted by phenol–chloroform extraction after bead-beating homogenization (FastPrep-24, MP Biomedicals) and purified using the Zymo Clean & Concentrator-5 Kit according to the manufacturer's instructions.

### 2.4. Quantitative PCR

Total RNA (1 μg/sample) was reverse-transcribed into cDNA using the High Capacity cDNA Reverse Transcription Kit (Thermo Fisher Scientific). Quantitative PCR was performed using the Universal ProbeLibrary system (Roche, Basel, Switzerland), with primers designed using the Roche online assay design tool. Reactions were run in duplicate using TaqMan Gene Expression Master Mix (Thermo Fisher Scientific) on an Applied Biosystems 7900HT Real-Time PCR System (Thermo Fisher Scientific). Relative mRNA levels were calculated using the 2^−ΔΔCt^ method and normalized to 18S rRNA. Primer and probe sets were: 18S_fwd: 5′ gca​att​att​ccc​cat​gaa​cg, 18S_rev: 5′ ggg​act​taa​tca​acg​caa​gc, 18S_probe: 48; Per2_fwd: 5′ tcc​gag​tat​atc​gtg​aag​aac​g, Per2_rev: 5′ cag​gat​ctt​ccc​aga​aac​ca, Per2_probe: 5; Bmal1_fwd: 5′ gcc​cca​ccg​acc​tac​tct, Bmal1_rev: 5′ tgt​ctg​tgt​cca​tac​ttt​ctt​gg, Bmal1_probe: 10; Reverba_fwd: 5′ cag​cat​gat​cag​gtc​aat​ctg​t, Reverba_rev: 5′ agc​aaa​tcg​tac​cat​taa​aac​ctc, Reverba_probe: 95.

### 2.5. RNA sequencing and analysis

Bulk RNA sequencing was performed on DRG tissue collected across a 52-hour circadian time series (14 time points at 4-hour intervals; n = 3 mice per time point). Equal amounts of RNA from the 3 biological replicates were pooled (2 µg total RNA) for library preparation using the TruSeq Stranded mRNA Library Prep Kit (Illumina, San Diego, CA). Sequencing was performed on an Illumina HiSeq platform with paired-end 50 bp reads, generating approximately 30 to 35 million paired-end reads per sample. Raw paired-end reads were filtered to remove ribosomal RNA contamination and aligned to the *Mus musculus* reference genome (GRCm39) using TopHat2 (v2.0.12),^39^ a splice-aware aligner built on Bowtie2. Gene annotations were obtained from Ensembl release 105 (GENCODE M28 equivalent). Alignments were performed for each time point, and resulting BAM files were coordinate-sorted using SAMtools (v0.1.19). Across the 14 libraries, an average of approximately 94.0% of QC-passed reads mapped to the reference genome (range, 92.9%–95.7%). Uniquely mapped reads averaged approximately 79.1% (range, 77.6%–81.1%). Gene-level expression was quantified using the Cufflinks suite (v2.2.1). Expression values were estimated as fragments per kilobase of exon per million mapped reads (FPKM) using Cuffdiff with bias correction and geometric normalization. Genes were retained for rhythmicity analysis if mean FPKM exceeded 0.5 across the time series after excluding zero-valued observations, reducing unstable phase estimates.

### 2.6. Circadian rhythmicity analysis

Circadian rhythmicity was assessed using multiple R algorithms. RAIN (Rhythmicity Analysis Incorporating Nonparametric methods)^74^ was used as the primary rhythm-detection method because it detects nonsinusoidal and asymmetric temporal profiles. Analyses were performed with a fixed 24-hour period, 4-hour sampling interval, independent method, and false-discovery correction; genes with adjusted RAIN q < 0.15 were considered rhythmic. This exploratory threshold was supported by complementary analyses using autoregressive spectral estimation and regression (ARSER), MetaCycle/JTK_CYCLE, and CircaCompare and by permutation testing.^32,42,74,78,82^ Because ARSER does not model replicate-level data, pooled time points were analyzed as a single 14-point profile per gene, and autoregressive spectral estimation (ARS)-derived phases were used downstream. Statistical significance was further evaluated by 1000 permutations in which circadian time labels were randomized while preserving data set structure.^70,71^ The empirical *P* value was calculated as (k + 1)/(N + 1), where k is the number of permutations yielding at least as many rhythmic genes as observed and N is the total number of permutations. Canonical clock genes (*Arntl*, *Clock*, *Npas2*, *Per1*, *Per2*, *Per3*, *Cry1*, *Cry2*, *Nr1d1*, and *Nr1d2*) were used to evaluate biological consistency across rhythm-detection methods.

### 2.7. Functional annotation and enrichment analysis

Rhythmic genes were stratified by peak circadian phase and grouped into phase bins for functional analysis. Gene Ontology enrichment was performed using the clusterProfiler package (v4.14.6) in R with annotations from org.Mm.eg.db (v3.20.0). Over-representation analysis was conducted using enrichGO with all expressed genes as the background universe. Multiple testing correction used the Benjamini–Hochberg method. Gene Ontology terms with adjusted *P* < 0.1 were considered enriched to detect broader phase-resolved trends across small gene sets unless otherwise stated; default clusterProfiler gene-set size filters were retained.^79,84^

Phase bins were defined using ARS-derived phase estimates. Circular variance was evaluated across multiple bin widths (2, 3, 4, and 6 hours) to determine optimal resolution. Although 2-hour bins showed the lowest variance, gene numbers were insufficient for stable enrichment analysis. Six-hour bins showed increased variance because of phase mixing. Three-hour bins provided the best balance between phase coherence and gene number and were used for all phase-resolved analyses.

### 2.8. Cell-type contextualization of rhythmic genes

To provide cellular context for rhythmic genes detected by bulk RNA-seq, rhythmic genes were mapped onto published mouse single-cell DRG reference atlases.^35^ For each rhythmic gene, mean normalized expression was calculated across major DRG cell classes: neurons, satellite glia, Schwann cells, fibroblasts, endothelial cells, pericyte/vascular cells, and macrophage/immune populations. Each gene was assigned to the class with the highest mean expression. Cell-type distributions were summarized as composition plots and *z*-scored heatmaps. This analysis was used to infer likely cellular sources of rhythmic genes rather than to claim cell type–specific rhythmicity.

## 3. Results

### 3.1. Spinal dorsal horn exhibits robust intrinsic circadian oscillations

To determine whether primary sensory pathways harbor intrinsic circadian rhythmicity, we examined the mouse cervical spinal cord using organotypic slices from *mPer2*^*luc*^ reporter mice. PER2-driven bioluminescence in transverse cervical spinal cord sections from postnatal (P7–P10) mice revealed self-sustained circadian oscillations. Rhythmic activity was detected in both dorsal and ventral horn regions, but amplitude was higher in the dorsal horn (Fig. 1A, B), the major site of somatosensory afferent input. Ventral horn regions exhibited lower-amplitude rhythms.

**Figure 1.**
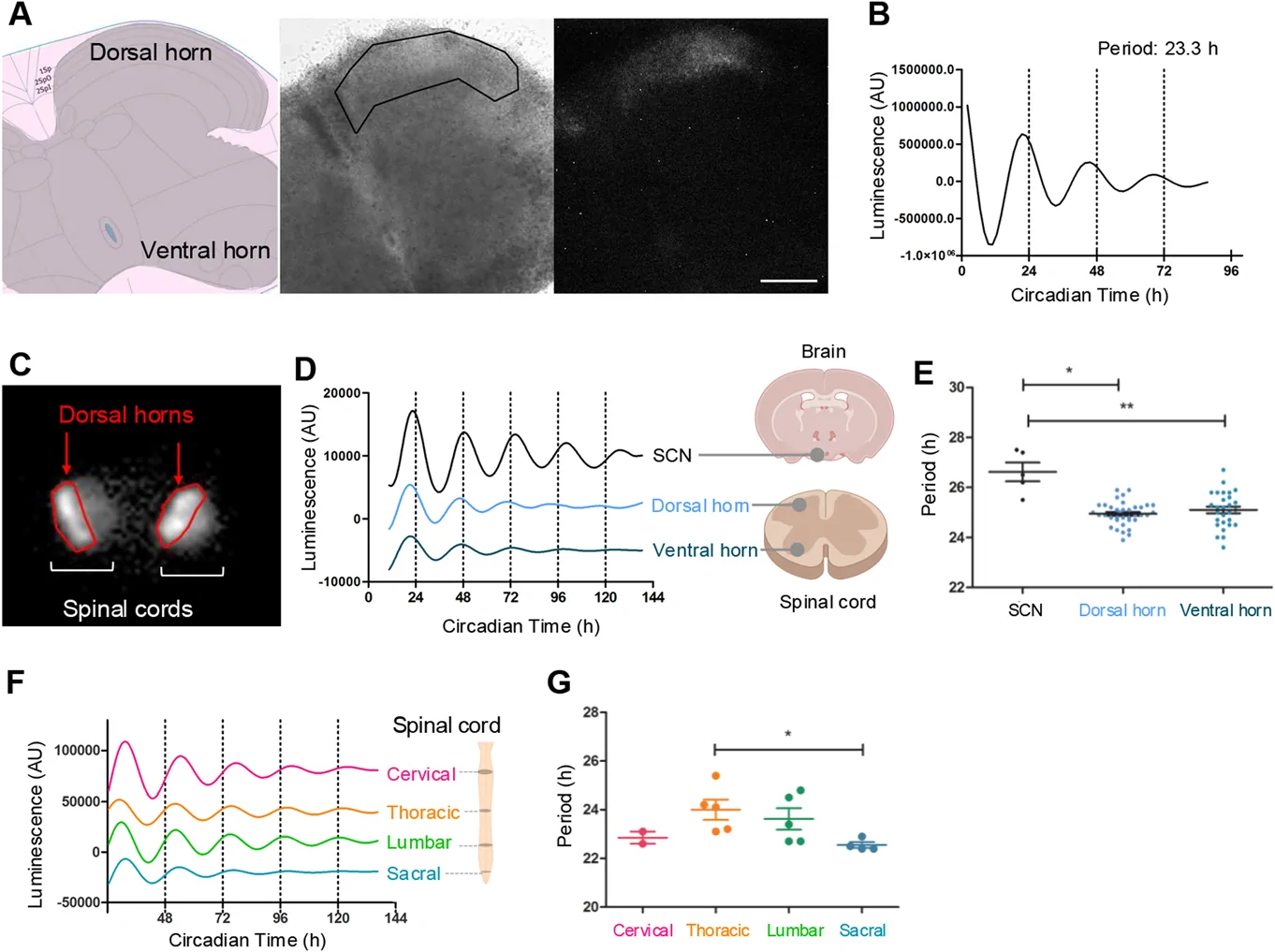
Autonomous PER2 circadian rhythms in the spinal cord dorsal horn. (A) Schematic transverse section of the mouse spinal cord at the C3 level (left), adapted from the Mouse Spinal Cord Atlas, illustrating gray matter (gray) and surrounding white matter (pink), with dorsal and ventral horns indicated. Representative brightfield image (middle) and corresponding bioluminescence image (right) of an organotypic cervical spinal cord slice from mPer2Luc mice. The outlined region marks laminae I-II of the dorsal horn. Scale bar, 200 μm. (B) Representative detrended PER2 bioluminescence trace (arbitrary units, AU) from the dorsal horn of a cervical spinal cord slice. (C) Macroscopic bioluminescence image of transverse spinal cord slices, with dorsal horn regions outlined in red. (D) Representative detrended PER2 bioluminescence traces from dorsal horn and ventral horn regions of spinal cord slices compared with SCN slices from the same animal. (E) Period of PER2 bioluminescence oscillations in SCN, dorsal horn, and ventral horn regions. Data are mean ± SEM with individual data points shown (n = 5 mice; n = 37 dorsal horn slices; n = 28 ventral horn slices). *P < 0.05, **P < 0.01 (Kruskal–Wallis test). (F) Representative detrended PER2 bioluminescence traces from dorsal horn regions of spinal cord slices at cervical, thoracic, lumbar, and sacral levels. (G) Period of PER2 bioluminescence oscillations across spinal levels. Data are mean ± SEM with individual data points shown. *P < 0.05 (Kruskal-Wallis test). SCN, suprachiasmatic nucleus.

We next compared spinal cord rhythms with those of the SCN. PER2-driven bioluminescence was measured in dorsal horn (n = 37 slices), ventral horn (n = 28 slices), and SCN (n = 5 slices) from the same cohort of animals (n = 5) (Fig. 1C). Under constant ex vivo conditions, spinal cord slices displayed autonomous circadian oscillations for multiple cycles. Compared with the SCN, oscillations in dorsal and ventral horn showed reduced amplitude and progressive damping (Fig. 1D), resembling rhythms in peripheral organs and extra-SCN brain regions.^1^

Despite amplitude differences, spinal cord rhythms maintained stable circadian periodicity (Fig. 1E). Intrinsic periods of dorsal horn and ventral horn oscillations were shorter than those of the SCN (24.95 ± 0.08 hours and 25.10 ± 0.14 hours, respectively, vs 26.62 ± 0.38 hours), consistent with the absence of specialized intercellular coupling mechanisms that lengthen and stabilize period in the SCN network.^48^

To assess rostrocaudal variation, we measured PER2 bioluminescence in dorsal horn slices from cervical, thoracic, lumbar, and sacral spinal levels. All regions exhibited self-sustained circadian oscillations, although amplitude varied across levels (Fig. 1F). By contrast, intrinsic circadian period differed only modestly, with thoracic segments showing slightly longer periods than sacral segments (Fig. 1G). These findings indicate conserved intrinsic clock function throughout the spinal dorsal horn.

### 3.2. Dorsal root ganglia exhibit autonomous circadian clock activity

We next asked whether circadian clock activity is also present in DRGs. Intact cervical, thoracic, and lumbar DRG explants exhibited robust PER2 bioluminescence rhythms under constant conditions (Fig. 2A, B). Intrinsic circadian periods did not differ significantly across spinal levels (cervical 24.67 ± 0.51 hours; thoracic 24.74 ± 0.24 hours; lumbar 24.52 ± 0.28 hours; *P* > 0.05), indicating conserved timing properties across DRGs.

**Figure 2.**
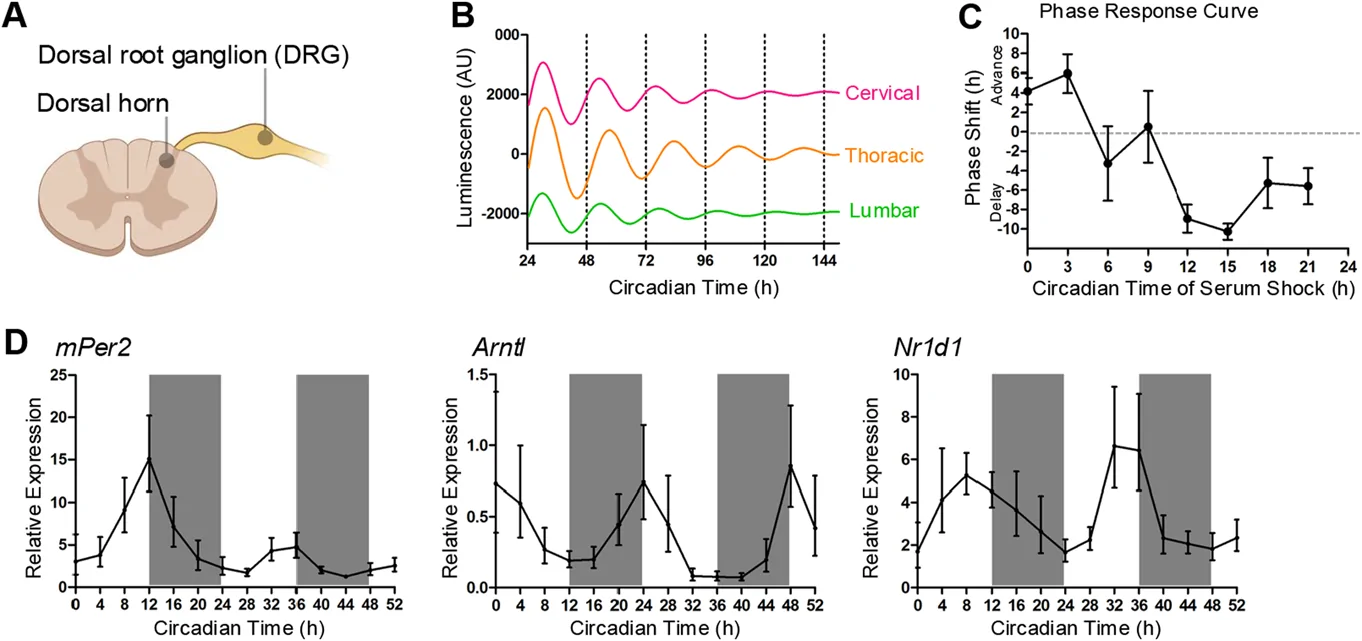
Autonomous circadian clock activity in the dorsal root ganglia. (A) Schematic of a transverse spinal cord section illustrating the dorsal horn and associated DRG. (B) Representative PER2 bioluminescence rhythms from individual *mPer2*^*Luc*^ DRG explants at cervical, thoracic, and lumbar spinal levels under constant ex vivo conditions. (C) Phase-response curve for DRG explants following serum shock applied at different circadian times. Phase advances are plotted as positive values and phase delays as negative values. (D) Quantitative RT-PCR analysis of key circadian clock gene expression (*mPer2*, *Arntl*, and *Nr1d1*) in DRGs collected across a 52-hour time course under constant darkness. Data represent mean ± SEM. DRG, dorsal root ganglion.

To determine whether DRG clocks can reset phase, we applied serum shock at different circadian times and quantified phase shifts. Phase resetting depended strongly on stimulus timing, producing a characteristic phase–response relationship (Fig. 2C). Serum shock early in the circadian cycle produced phase advances of up to ∼6 hours, whereas stimulation at the opposite phase induced delays of up to ∼10 hours.

To confirm that circadian rhythms are present in vivo, we collected DRGs across a 52-hour time course in constant darkness and measured key clock gene expression by quantitative PCR. *mPer2*, *Arntl* (*Bmal1*), and *Nr1d1* exhibited robust rhythmic expression (Fig. 2D), with *mPer2* and *Arntl* oscillating in antiphase as expected.^58^ These findings show that DRGs harbor self-sustained circadian clocks that operate autonomously ex vivo and persist in vivo.

### 3.3. RNA-seq reveals phase-structured circadian gene expression in dorsal root ganglia

To determine whether circadian regulation extends broadly across the DRG transcriptome, we performed RNA-seq on DRGs collected across a 52-hour circadian time course in constant darkness (CT0-52; n = 3 mice/time point; Fig. 3A). RAIN analysis identified 626 genes with significant circadian rhythmicity (q < 0.15), representing 3.6% of the 17,394 expressed genes detected in DRGs (Supplementary File 1, https://links.lww.com/PR9/A450). Permutation analysis confirmed that this exceeded chance expectation (empirical *P* = 0.000999; Supplementary Figure 1A, https://links.lww.com/PR9/A450). Secondary analyses showed consistent support for the canonical clock network across algorithms (Supplementary Figure 1B, C; Supplementary File 2, https://links.lww.com/PR9/A450). Canonical clock genes were strongly enriched among rhythmic genes (Fisher exact test: *P* = 1.15 × 10^−10^; odds ratio (OR) = 108.13; 95% confidence interval (CI) = 21.53–1023.28; Fig. 3B). Representative clock genes, including *Arntl* and *Per2*, showed expected antiphasic pattern (Fig. 3C)^58^; additional clock-gene trajectories are shown in Supplementary Figure 1D (https://links.lww.com/PR9/A450).

**Figure 3.**
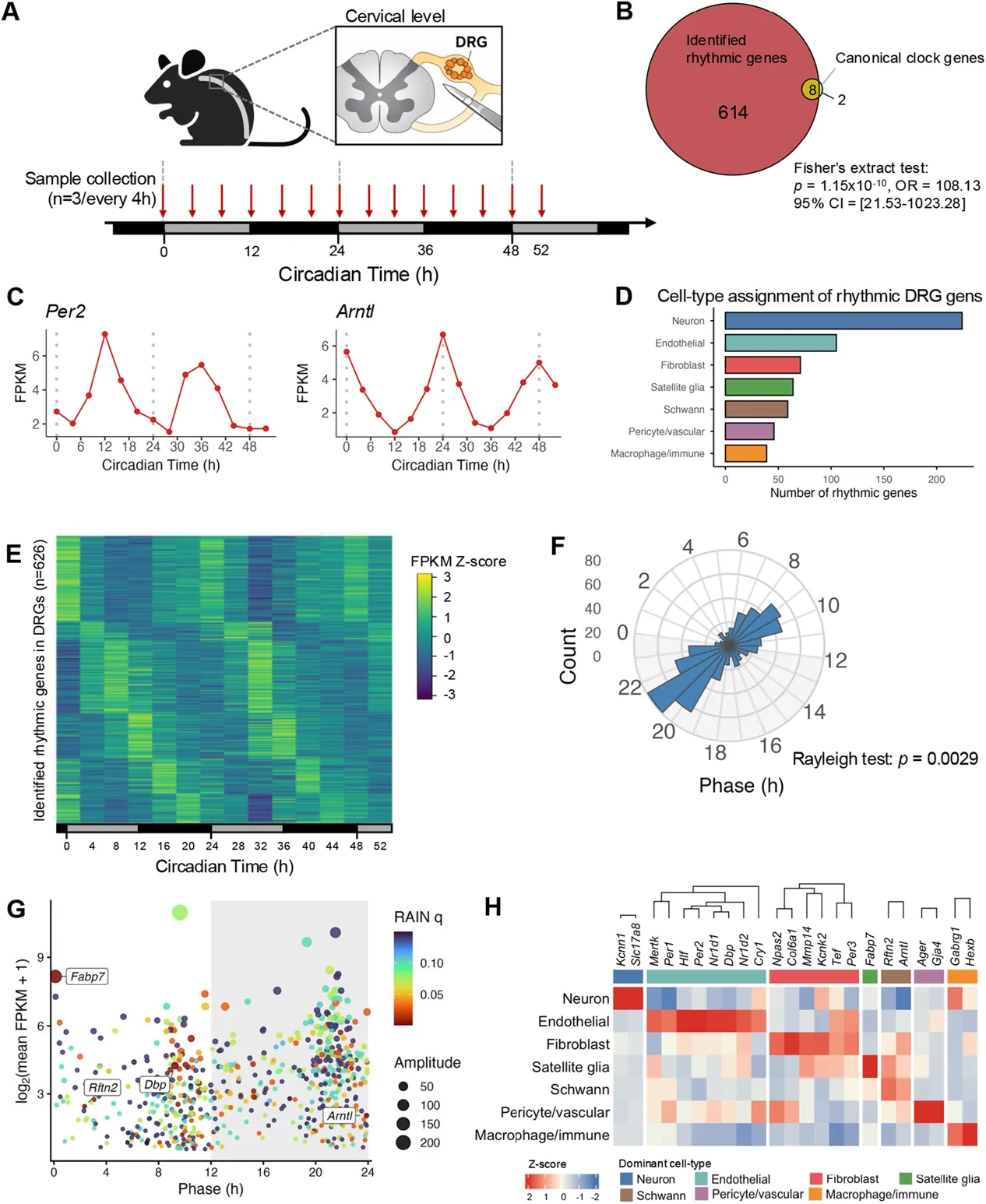
Comprehensive identification of circadian genes in dorsal root ganglia. (A) Schematic of the experimental design for circadian transcriptomic profiling of mouse dorsal root ganglia (DRGs). Adult mice were maintained under constant darkness (DD), and DRGs were collected every 4 hours across a 52-hour circadian time course (CT0-CT52; n = 3 mice per time point). The timeline indicates circadian time, with white and black bars denoting subjective day and night, respectively. DRG dissection from the spinal column is illustrated in the inset. (B) Enrichment of canonical clock genes among rhythmic DRG genes. Of the 626 circadian genes identified by RAIN (q < 0.15), 8 of 10 core clock genes (highlighted in yellow) were detected, significantly exceeding chance expectation (Fisher exact test: *P* = 1.15 × 10^−10^; OR = 108.13), indicating strong overrepresentation of clock components within the rhythmic gene set. (C) Representative expression profiles of *Arntl* and *Per2* showing characteristic antiphase oscillations across circadian time. (D) Cell-type contextualization of rhythmic DRG genes using a published mouse single-cell DRG atlas. Each rhythmic gene was assigned to a dominant cell class based on the highest average expression across major DRG cell types. (E) Heatmap of *z*-scored expression profiles for RAIN-identified rhythmic genes in DRGs (n = 626), ordered by peak circadian phase to highlight temporal structure. (F) Circular histogram showing the distribution of peak circadian phases for rhythmic DRG genes identified by RAIN analysis (q < 0.15). Phase clustering was nonuniform as assessed by the Rayleigh test for circular uniformity (*P* < 0.01), indicating organized temporal regulation. Phases were derived from ARS-adjusted phase estimates and binned at 1-hour resolution across the 24-hour circadian cycle; the radial axis denotes gene count per phase bin. (G) Phase-expression plot of rhythmic DRG genes. Each point represents a rhythmic gene identified by RAIN (q < 0.15), plotted by peak circadian phase (*x*-axis) and mean gene expression [log_2_(mean FPKM + 1); *y*-axis]. Point color indicates RAIN q value, and point size represents oscillation amplitude. Gray shading denotes subjective night (CT12–24). Selected genes with strong rhythmicity are labeled. (H) Heatmap showing relative enrichment of selected rhythmic genes across major DRG cell types derived from single-cell atlas data. Expression values are log-transformed and *z*-score normalized across cell types, and genes are grouped by dominant cell-type assignment.

Because DRG tissue contains diverse neuronal and non-neuronal populations, we mapped rhythmic genes onto a published mouse single-cell DRG atlas. Rhythmic genes were broadly distributed across ganglionic cell types rather than confined to neurons (Fig. 3D). Of the rhythmic genes represented in the atlas, 224 (36.8%) mapped most strongly to neurons, whereas 63.2% mapped to non-neuronal populations, including endothelial cells (105 genes; 17.3%), fibroblast-like cells (71; 11.7%), satellite glia (64; 10.5%), Schwann cells (59; 9.7%), pericyte/vascular cells (46; 7.6%), and macrophage/immune cells (39; 6.4%).

Hierarchical clustering of *z*-scored expression profiles showed clear temporal structure across the circadian cycle (Fig. 3E). Peak-phase analysis revealed a nonuniform distribution, with 262 genes (41.9%) peaking during the subjective day and 364 (58.1%) during the subjective night. This distribution was significantly nonrandom (Rayleigh test: *P* = 0.0029; Fig. 3F), indicating that rhythmic genes assemble into distinct circadian phase windows.

Plotting rhythmic genes in phase-expression space showed a broad range of transcript abundance and oscillation amplitude (Fig. 3G), indicating that rhythmicity is not restricted to highly expressed genes. Clock-associated genes such as *Arntl* and *Dbp* peaked at expected phases.^58,86^ Among the strongest rhythmic genes, *Fabp7*^11^ showed high amplitude and strong significance, whereas *Rftn2*^21^ also exhibited clear rhythmicity with an early-day peak phase. Atlas-based visualization of representative rhythmic genes further showed distinct cellular enrichment patterns across DRG cell classes (Fig. 3H), including neuronal enrichment of several ion channel and clock-related genes and non-neuronal enrichment of *Fabp7* and *Rftn2*. These findings indicate that circadian gene expression in DRGs reflects a ganglion-wide, phase-structured program spanning multiple cell classes.

### 3.4. Circadian phase stratification reveals functional organization of rhythmic dorsal root ganglia genes

To determine whether rhythmic genes are organized into phase-specific biological programs, we performed Gene Ontology analysis on rhythmic genes stratified by peak phase (Fig. 4A; Supplementary File 3, https://links.lww.com/PR9/A450). Genes peaking during early subjective day (CT0-3) were enriched for organic acid and carboxylic acid transport (Fig. 4B, C), pathways relevant to neuronal metabolism and signaling.^75^

**Figure 4.**
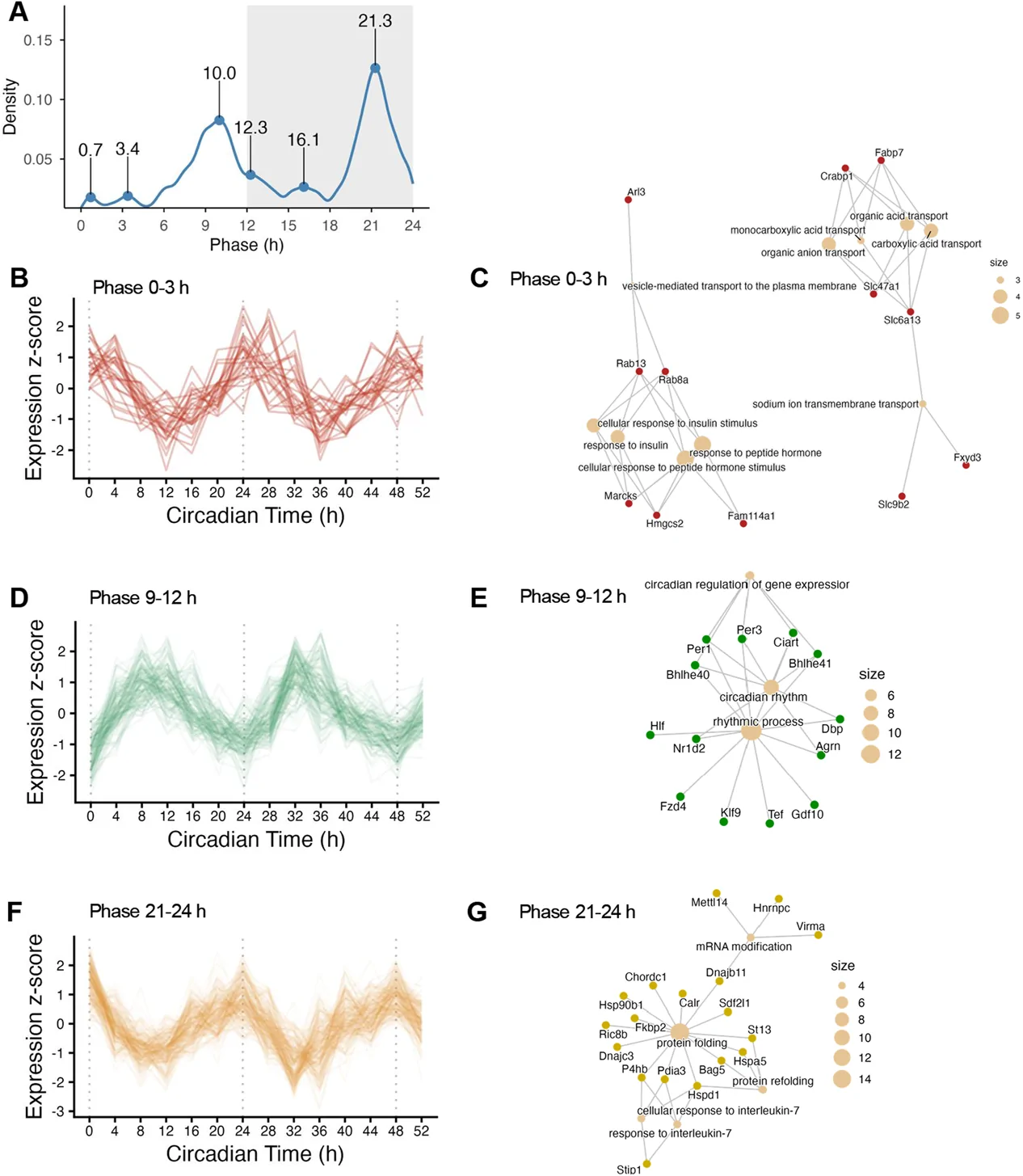
Circadian transcription in dorsal root ganglia (DRGs) is organized into discrete phase-specific gene expression programs with distinct biological functions. (A) Kernel density estimate of peak circadian phases for rhythmic DRG genes identified by RAIN (q < 0.15). Local maxima indicate preferred phase windows, supporting stratification into discrete 3-hour phase bins. (B, D, F) *Z*-score-normalized expression trajectories of rhythmic genes peaking at CT0-3 (B), CT9-12 (D), and CT21-24 (F). Each line represents an individual gene; dotted vertical lines denote 24-hour intervals. (C, E, G) Gene Ontology biological process (GO-BP) enrichment networks for genes within the corresponding phase bins shown at left. Nodes represent enriched GO terms (beige) and associated genes (colored), with node size proportional to gene count per term. Terms meeting an exploratory enrichment threshold (BH-adjusted *P* < 0.1) are shown.

During mid-day phases (CT3-6 and CT6-9), rhythmic genes were enriched for processes associated with neuronal structure and differentiation, including dendritic spine morphogenesis and neuron projection development (Supplementary Figure 2A–D, https://links.lww.com/PR9/A450). During the late day transition (CT9–12), genes were enriched for circadian regulation of gene expression and rhythmic processes (Fig. 4D, E). By contrast, genes peaking during the subjective night showed less coherent functional annotation overall. Early night (CT12–15) showed amino acid transport (Supplementary Figure 2E, F, https://links.lww.com/PR9/A450), whereas genes peaking late in the night (CT21–24) were enriched for protein folding, protein refolding, and cellular stress–response pathways (Fig. 4F, G). This late phase represents a proteostatic and RNA-regulatory program. These findings indicate that the circadian DRG transcriptome is organized into discrete, phase-specific functional programs rather than a uniform oscillatory output.

Baseline circadian organization reveals functional stratification of neuropathic pain-associated genes in DRGs.

To ask whether baseline circadian architecture reveals additional structure in pain-relevant gene programs, we cross-referenced rhythmic DRG genes with genes associated with spared nerve injury (SNI)–induced neuropathic pain in DRGs^18^ and with neuropathic pain-related signaling pathways from PathCards (Fig. 5A–C). Only a small subset overlapped the rhythmic set, and enrichment testing did not detect significant overrepresentation of rhythmic genes within neuropathic pain-associated datasets.

**Figure 5.**
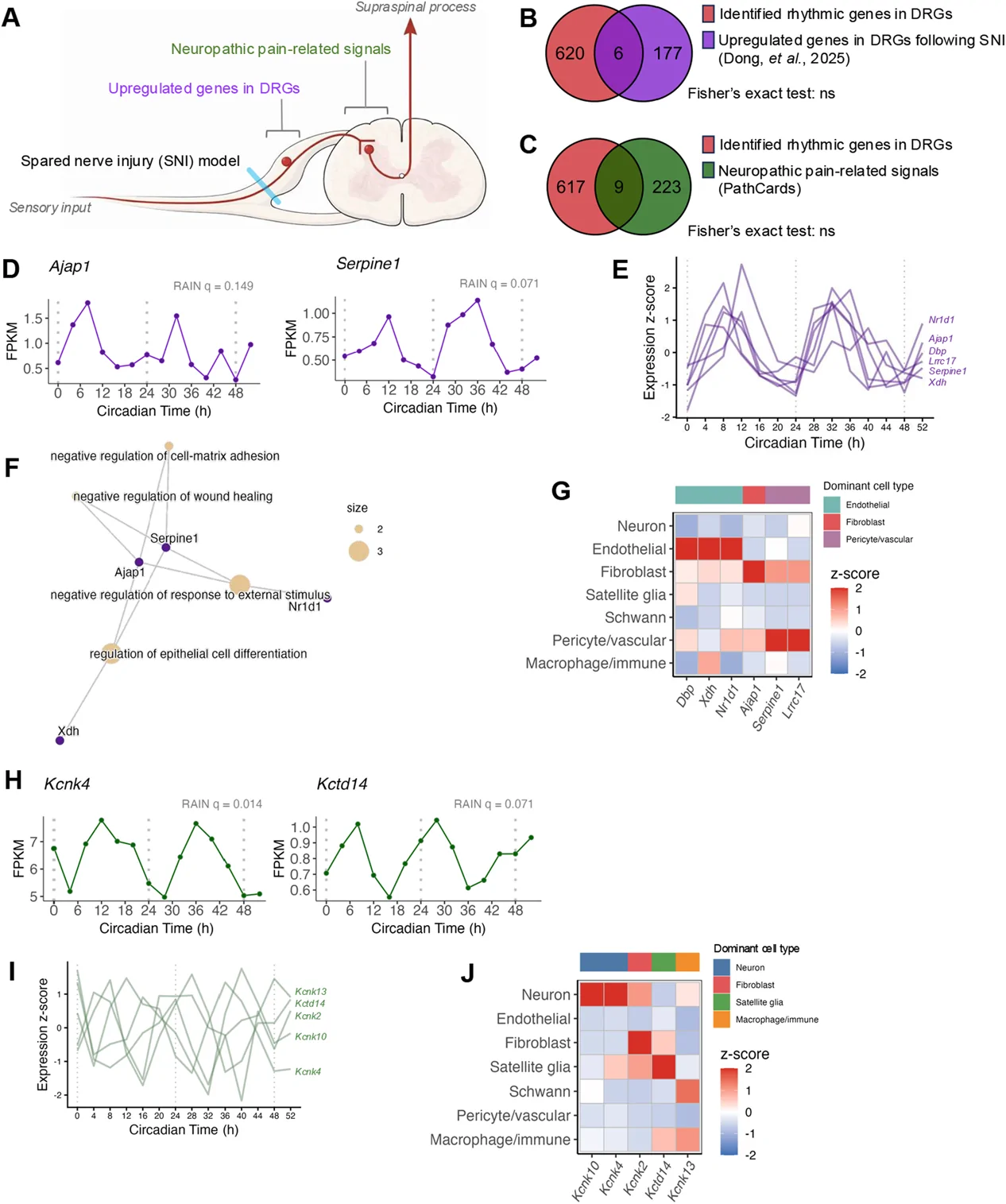
Baseline circadian organization reveals functional stratification of neuropathic pain-associated gene programs in DRGs. (A) Conceptual schematic of the SNI model highlighting the sensory neuron-DRG–spinal cord axis. Neuropathic pain-related signaling pathways (green) and genes reported to be upregulated in DRGs after nerve injury (purple) are shown in relation to ascending supraspinal processing, providing anatomical context for integration with baseline circadian gene expression programs. (B) Venn diagram showing overlap between circadian rhythmic genes identified in DRGs under baseline conditions (RAIN q < 0.15; red) and genes upregulated in DRGs following neuropathic pain in published SNI datasets (purple; Dong et al., 2025). Only a small subset of genes overlapped (n = 6; Fisher exact test, not significant), indicating minimal enrichment of injury-induced genes among baseline circadian genes. (C) Venn diagram showing overlap between baseline circadian rhythmic DRG genes (RAIN q < 0.15; red) and neuropathic pain-related signaling genes curated from PathCards for dorsal horn neurons (green). As in panel B, overlap was limited (n = 9; Fisher exact test, not significant). (D) Representative baseline time-course expression profiles of overlapping rhythmic genes (*Ajap1* and *Serpine1*) in DRGs under constant darkness. (E) *Z*-score-normalized baseline expression profiles of all overlapping rhythmic genes across the circadian cycle, illustrating convergence onto a shared circadian phase window. (F) Gene Ontology enrichment network for overlapping rhythmic genes, highlighting pathways related to tissue remodeling and regulation of injury responses (*P* < 0.05, q < 0.05). (G) Cell-type contextualization of overlapping rhythmic genes using a published mouse single-cell DRG atlas. Expression values were log-transformed and *z*-score normalized across major DRG cell classes, and genes were grouped by dominant cell-type assignment. (H) Representative baseline time-course expression profiles of neuropathic pain-associated potassium channel-related genes (*Kcnk4* and *Kctd14*) identified from dorsal horn-centered pathway annotations. (I) *Z*-score-normalized baseline expression profiles of potassium channel-related genes (*Kcnk2*, *Kcnk4*, *Kcnk10*, *Kcnk13*, and *Kctd14*) across the circadian cycle, showing circadian modulation without clear phase convergence. (J) Cell-type contextualization of potassium channel-related genes using a single-cell DRG atlas, showing relative enrichment across major DRG cell classes. DRGs, dorsal root ganglia; SNI, spared nerve injury.

Among overlapping genes, *Ajap1* and *Serpine1*, both of which have been implicated in regenerative or repair-associated processes after injury,^67^ exhibited circadian variation under baseline conditions (Fig. 5B, D). Their temporal profiles aligned to a shared phase window (Fig. 5E), and Gene Ontology analysis highlighted pathways related to tissue remodeling and injury responses (Fig. 5F). Mapping these genes onto a mouse DRG single-cell atlas showed distinct cellular enrichment across ganglionic cell classes (Fig. 5G).

By contrast, comparison with neuropathic pain-related signaling pathways identified several potassium channel-related transcripts, including *Kcnk4*, *Kcnk10*, *Kcnk13*, and *Kctd14*, that also showed circadian variation in DRGs (Fig. 5C, H, I). Unlike remodeling-associated genes, these transcripts did not show strong phase convergence, indicating gene-specific temporal regulation within this functional group. This pattern is consistent with the functional diversity of potassium channel subtypes, which differ in their biophysical properties, regulatory mechanisms, and contributions to sensory neuron excitability.^20,30,56^ Atlas mapping showed distinct cellular expression patterns across neuronal and non-neuronal populations (Fig. 5J). These data indicate that neuropathic pain-associated genes intersect baseline circadian organization in a function-dependent manner: remodeling-related genes align to shared phase windows, whereas excitability-related genes show heterogeneous timing.

## 4. Discussion

Our findings indicate that spinal dorsal horn and DRGs harbor self-sustained circadian clocks and that DRG gene expression is organized into phase-resolved transcriptional programs. These results support a model in which intrinsic clocks generate daily windows of cellular preparedness.

Circadian function in spinal cord and DRGs has remained incompletely characterized. Although circadian rhythms in these tissues have been implicated in pain, restless legs syndrome, and autoimmune disorders,^6,13,28,81^ much of the evidence comes from studies performed under LD conditions, making it difficult to separate intrinsic regulation from environmental or behavioral influences. By combining long-term ex vivo bioluminescence imaging with DRG transcriptomic profiling under constant conditions, we show that both spinal dorsal horn and DRGs harbor tissue-autonomous circadian clocks. To the best of our knowledge, this is the first real-time characterization of PER2 rhythms in these sensory tissues together with a comprehensive circadian transcriptome of adult DRG.

Although clock gene expression has previously been reported in spinal cord, the pronounced rhythmic amplitude observed in dorsal horn is notable. Dorsal horn regions, where primary afferent input is received and integrated, showed stronger circadian amplitude than ventral motor regions. Circadian oscillations have also been described in spinal astrocytes, particularly at lumbar levels, and linked to motor and autonomic regulation.^54,72^ Our data extend these observations to sensory-associated spinal compartments.

A central advance of this study is the phase-structured DRG transcriptome. Although only 3.6% of expressed genes met the rhythmicity threshold, this proportion is comparable with previous bulk circadian transcriptomic studies of the nervous system, where cellular heterogeneity is believed to attenuate rhythmic signals through averaging across diverse neuronal and non-neuronal populations.^55,86^ Nevertheless, rhythmic genes were distributed nonuniformly across circadian phase and assembled into distinct temporal clusters. Gene Ontology enrichment within these clusters revealed coherent biological programs, arguing against a single global circadian output and supporting multiple temporally separated transcriptional states.

Genes peaking early in the subjective day were enriched for organic and carboxylic acid transport, suggesting circadian regulation of metabolic substrate handling and signaling capacity. *Fabp7* has recognized roles in lipid trafficking and neuroinflammatory signaling and has been implicated in persistent pain states through immune-glial interactions.^77^ *Slc6a13* (GAT-3), a GABA transporter, may link circadian transcription to longer-term regulation of inhibitory tone and metabolite flux in neural tissue.^59^

Mid-day phases were enriched for growth factor signaling, extracellular matrix organization, and structural remodeling. Genes including *Igfbp5, Comp, Col6a1, Bmp6, Fbn1,* and *Fzd7* are linked to ECM dynamics and regenerative signaling, processes relevant to nerve injury responses and persistent pain.^10,24,46,52^ Wnt signaling has been shown to sensitize peripheral sensory neurons and contribute to pain hypersensitivity.^69^ Other rhythmic genes in these clusters, including *Tiam1, Ngef, Il1rapl1, Nfatc4*, and *Abi3bp* likely reflect shared molecular machinery involved in cytoskeletal regulation and neurite remodeling.^3,23,44,45,47^ Rhythmic expression of these genes may therefore mark circadian windows during which structural remodeling or regenerative processes are transcriptionally favored.

The most prominent night-associated enrichment occurred late in the cycle, with strong representation of protein folding and ER chaperone pathways, including *Hspa5/GRP78, Hsp90b1/GRP94, Calr, Pdia3, P4hb, Dnajc3*, and cochaperones.^51,60^ ER stress responses in DRGs have been implicated in neuropathic pain and mechanical hypersensitivity.^33,85^ This phase also contained genes involved in RNA processing and modification, including *Mettl14, Virma/KIAA1429, Hnrnpc*, suggesting that circadian organization in DRGs extends beyond transcription to post-transcriptional regulation.^49^

From a translational perspective, these findings provide a molecular framework for understanding why pain states and treatment responses can vary by time of day. Rather than implying that circadian clocks acutely gate nociception, our data support a model in which circadian programs bias the baseline physiological state of sensory tissues through metabolic regulation, structural remodeling, proteostasis, and RNA processing, thereby influencing responses to inflammation, nerve injury, or repeated nociceptive input.

Cross-referencing circadian transcripts with neuropathic pain-associated data sets revealed limited overlap overall. However, genes such as *Ajap1*, *Serpine1*, *Lrrc17*, and *Xdh,* aligned with circadian phases enriched for tissue remodeling programs. *Serpine1* regulates plasmin-mediated extracellular matrix proteolysis and increases after nerve injury.^57,67,68^ The clock gene *Bmal1* has also been reported to gate axon regeneration in DRG neurons, suggesting direct clock control of this process.^16,26^ *Ajap1* has been linked to Schwann cell myelination, leucine-rich repeat-containing proteins to nociceptive circuit maintenance, and *Xdh* to redox metabolism in sensory physiology.^4,5,17,31,66^ Together, these genes suggest a circadian window in which structural maintenance and regenerative programs are transcriptionally engaged under baseline conditions.

Potassium channel–related genes identified in neuropathic pain signaling pathways also showed baseline circadian modulation in DRGs. Because potassium channels are major regulators of neuronal excitability and sensory transmission, which differ in their electrophysiological properties and regulatory mechanisms, their gene-specific temporal patterns may represent an additional mode by which circadian timing shapes sensory state.^9,14,22,73^

Several limitations should be noted. Dorsal root ganglia differ anatomically and functionally along the rostrocaudal axis, and our transcriptomic analysis pooled ganglia at the cervical level, which may obscure level-specific or cell type–specific rhythms. Rhythmic mRNA does not necessarily imply rhythmic protein abundance, and rapid changes in excitability are unlikely to be driven solely by transcriptional oscillations.^62,64^ In addition, our tissue-level bioluminescence recordings do not resolve synchrony among individual cells or coordination between DRG and dorsal horn clocks in vivo. Future work combining single-cell imaging, spatial transcriptomics with proteomic or phosphoproteomic profiling, sex-balanced experimental designs and testing phase-dependent responses in injury models, will be required to define the underlying mechanisms. Temperature is also a major entrainment cue for peripheral clocks.^7^ Although we did not directly test temperature entrainment in DRGs, previous studies implicate HSF1 and cold-induced RNA-binding proteins in temperature-dependent clock resetting.^65^ Because DRGs lie outside the blood–brain barrier and experience local thermal fluctuations, temperature-driven entrainment may be physiologically relevant in peripheral sensory tissues.

## Disclosures

The authors have no conflict of interest to declare.

## Supplemental digital content

Supplemental digital content associated with this article can be found online at https://links.lww.com/PR9/A450.
